# Longitudinal performance trajectories of young female sprint runners: a new tool to predict performance progression

**DOI:** 10.3389/fspor.2024.1491064

**Published:** 2024-12-23

**Authors:** Michael Romann, Marie Javet, Julia Hernandez, Louis Heyer, Severin Trösch, Stephen Cobley, Dennis-Peter Born

**Affiliations:** ^1^ Department of Elite Sport, Swiss Federal Institute of Sport Magglingen, Magglingen, Switzerland; ^2^ Faculty of Science and Medicine, University of Fribourg, Fribourg, Switzerland; ^3^ Swiss Athletics Federation, Ittigen, Switzerland; ^4^ Datahouse, Zürich, Switzerland; ^5^ Exercise & Sport Science, Faculty of Health Sciences, The University of Sydney, Sydney, NSW, Australia; ^6^ Swiss Development Hub for Strength and Conditioning in Swimming, Swiss Aquatics—National Swimming Federation, Worblaufen, Switzerland

**Keywords:** talent identification & development, benchmarking, performance prediction, female athlete, sprinting

## Abstract

**Background:**

Longitudinal performance tracking in sports science is crucial for accurate talent identification and prognostic prediction of future performance. However, traditional methods often struggle with the complexities of unbalanced datasets and inconsistent repeated measures.

**Purpose:**

This study aimed to analyze the longitudinal performance development of female 60 m sprint runners using linear mixed effects models (LMM). We sought to generate a practical tool for coaches and researchers to establish benchmarks and predict performance development.

**Methods:**

We analyzed 41,123 race results from 8,732 female 60 m track sprinters aged 6–15 years, collected from the Swiss Athletics online database between 2006 and 2021. Only season-best times per athlete and only athletes with at least 3 season-best times in their career were included. LMM was used to generate performance trajectories, benchmarks, and individual predictions. A practical software tool was developed and made available to allow individual performance prediction based on race times from previous seasons. In addition, classic empirical percentile curves were constructed using the Lambda-Mu-Sigma (LMS) method.

**Results:**

LMM handled the dataset's complexities, producing robust longitudinal performance trajectories. Compared to empirical percentiles generated using the LMS method, which provided a retrospective view of performance development, the mixed model approach identified individualized longitudinal performance developments and estimated predictions of future performance. The best-fitting model included log-transformed chronological age (CA) as a fixed effect and random intercepts and slopes for each athlete. This model explained 59% of the variance through fixed effects (marginal R^2^) and 93% through combined fixed and random effects (conditional R^2^).

**Conclusion:**

LMM provided longitudinal sport performance data, enabling the establishment of performance benchmarking and prediction of future performance. The software tool can assist coaches in setting realistic training goals and identifying promising athletes.

## Introduction

Understanding the individual athlete's journey from initiation to peak performance is of fundamental interest to athlete development researchers and sport practitioners. The trajectory of an athlete's performance is influenced by a multitude of contextual, environmental, and individual factors, as highlighted in recent studies on talent identification and talent development in various sports. Understanding these factors is critical for designing effective athlete development frameworks and tailoring interventions to optimize long-term performance outcomes (1, 2). A recent and growing body of research highlights the importance of assessing performance development, rather than relying solely on current competitive performance, as a critical criterion for sustainable and long-term talent development. To understand development, longitudinal data assessments are required rather than a single performance test (1, 3, 4). At an individual level, performance development is influenced by contextual, environmental, and training conditions, anthropometric and physiological development, as well as biomechanical and technical skill development. This complexity complicates athlete assessment and makes predictions at a young age difficult (5). Multi-factorial and regular assessments, as provided by longitudinal data collection, are required to establish a comprehensive understanding of performance development, i.e., baseline starting points, relative progress and change, rates of development relative to other athletes, and the likely underlying factors contributing (or not contributing) to progression (or sub-optimal progression) (6). As such, there is a growing interest in determining practical developmental benchmarks (7) and performance forecasting based on longitudinal data, as these methods optimise the use of existing resources to improve athlete development and gain a competitive advantage (8–10).

Benchmark percentiles can serve several purposes in athlete development: talent selection, identification of progressing latent talent, organizational/coaching interventions and programme targeting (11). Performance trajectories can also be used to construct percentile curves, which determine an individual's relative progress at a given point in development (or over a period of time) compared to a specific reference population. There are two main methods for constructing percentile curves: empirical percentiles—calculated directly from observed data using the Lambda-Mu-Sigma (LMS) method—and mixed models. The LMS method models the changing distribution of measurements over time and includes three parameters: Lambda (L), Mu (M), and Sigma (S), which represent the skewness, the median, and the coefficient of variation, respectively. These parameters are fitted with a maximum penalized likelihood, to smooth the percentile curves, which is particularly useful when assessing individual trajectories relative to a specific reference population (12–14). However, the accurate estimation of percentiles from the LMS method relies on the assumption that the variables of interest are normally distributed after the transformation and smoothing. Although this method has been widely used in fields such as health and sport science to produce reference growth charts and to monitor physical fitness (15), the LMS method primarily provides a retrospective view of past performance development and may not adequately account for individual variability over time in a longitudinal dataset. As such, when analyzing longitudinal performance the mixed model approach is superior to the LMS, due to its flexibility when dealing with unbalanced data, its ability to provide individualized trajectories and predictions, its statistical robustness, and its greater explanatory power (16).

Linear mixed models (LMMs) are particularly well suited to dealing with the complexities inherent to longitudinal sports data, such as multiple dependent observations and unbalanced data. These models account for the heterogeneity in the frequency of observations due to factors such as injuries, team selection and changes in competition schedules, making them ideal for longitudinal sports science research (16–18). This flexible statistical approach incorporates both fixed and random effects allowing a comprehensive analysis of performance trajectories and allows researchers to include multiple predictors and account for individual differences while improving the accuracy of longitudinal trends and allowing for benchmarking. An additional advantage of the LMM is the ability to predict and forecast future trajectories (e.g., performance development) based on a combination of current and past information (10, 16, 19). LMM-predictions are calculated by combining the best linear predictor of random effects with the best linear estimate of fixed effects. Typically, predictions are made for a subset of explanatory variables at given values, while the remaining variables are either averaged or set to specific values. The prediction process involves selecting explanatory variables and relevant model terms, determining averaging variables, and deciding on appropriate weightings for the averaging across dimensions in the prediction model (10, 19). If accurate, such predictive capabilities could beneficially inform the decision-making and programming of sports scientists and practitioners. For example, Born et al. (11) demonstrated the utility of LMM in the development of normative data and percentile curves for long-term athlete development in swimming, demonstrating their effectiveness in establishing cohort-based performance benchmarks and individualized predictions. In addition, Antink et al. (20) highlighted the value of longitudinal data in formulating more accurate predictions of future athlete performance decline in Swedish veteran track and field athletes.

Taken together, performance trajectories and predictions of future performance can improve athlete and talent development. This study addresses the challenge of providing individualized performance predictions in youth athletics by leveraging advanced longitudinal modeling techniques. Unlike traditional percentile-based methods, this research integrates individual performance developments through linear mixed-effects models to establish benchmarks and forecast future performance, offering a novel tool for coaches and practitioners to support talent development. By generating a practical tool for practitioners, athlete assessment, individual athlete development programming, coaching intervention, and talent selection could be improved.

The primary aims of the study were: (1) to establish age-specific reference values using classical empirical percentile curves and progressive mixed model approaches, and (2) to develop a prediction model and a practical software tool to predict future performance development.

## Methods

### Subjects

To analyze race results from female 60 m sprint runners from competitions held in Switzerland between 2006 and 2021, a total of 160,852 observations were provided by the online and public database of the Swiss Athletics Federation. Only results from officially licensed outdoor competitions were selected for this study. In addition, only results for athletes aged between 6 and 15 (11.3 ± 2.1 years) have been retained in the database (observations *n* = 160,667). This was justified because the 60 m sprint is an official distance in Switzerland only up to U16. After that, the official distance is 80 m. All data were analyzed anonymously. The study was approved by the institutional review board of the Swiss Federal Institute of Sport Magglingen (Reg.-Nr. 227-2024) and by the ethical standards of the World Medical Association (Declaration of Helsinki). No written informed consent from the subjects was required, as the present study utilized only publicly available data that were analyzed anonymously.

### Procedure and data analysis

To ensure a high quality of the data, results that deviated by more than 3 standard deviations from the average performance within each age category were identified as outliers and removed from further analysis. To be included in the data set for the longitudinal data analysis, only athletes with at least three seasons of participation (minimum for longitudinal analysis) were further considered (21). The season's best times were used to identify trends and development patterns over time. According to the later described statistical model, the three race results did not have to originate from three consecutive seasons. The final data set contains 41,123 observations from 8,732 different female athletes. All data analyses were completed using R statistical software (R Core Team) version 2024.04.2 + 764.

### Empirical percentile curves vs. linear mixed model approach

#### Empirical percentile curves

To achieve the first aim of this study, which is to establish age-related reference values, two methods were analyzed. The first method, already used in sports science for modeling smoothed percentile curves, is the LMS method (11, 15). The LMS method is particularly effective for modeling growth and performance data as it accounts for skewness and kurtosis in the distribution (13). The GAMLSS (Generalized Additive Models for Location, Scale, and Shape) method was used to fit the LMS models, utilizing the gamlss function from the R package of the same name (22). This method provides a high flexibility, which is crucial for capturing complex variations in athletic performance across different ages. Only the 3rd, 10th, 25th, 50th, 75th, 90th and 97th percentile curves were then plotted for visual representation.

#### Mixed model approach

As a second method, linear mixed effects model (LMM) was used. LMM enables the analysis of longitudinal measures with a variable number of observations per subject without excluding data, in contrast to more traditional methods such as ANOVA. Furthermore, LMM is particularly well suited to explaining the development over time (16). To generate the reference values with the LMM, several steps were undertaken. First the predictive model explaining the relationship between sprint performance and chronological age (CA) was created. For that, a new variable CA_mindiff’ (later called CAdiff) was calculated CAdiff = CA- min (CA) + 1, resulting in an *x*-axis intersection at one.

Then normal distribution of the data was investigated with a Q-Q plot and Kolmogorov tests. Both tests indicated non-normally distributed data (*p* < 0.05). The data were log-transformed to compensate for the non-normal distribution and to linearize the relationship between performance and CA.

In the next step of the LMM analysis, the best fitting mixed effects model was identified. Models were formulated using the LMER (linear mixed effects regression model) function from the lme4 (v1.1-31) package in R Studio (23). The initial model, with logarithmic sprint performance as the dependent variable, includes one overall fixed intercept and random intercepts for each athlete (represented by id). This model accounts for the variability in results across athletes who appear multiple times in the data frame and serves as the base model. Then stepwise forward variable selection was done to define successive models. The second model additionally introduces logarithmic age as a fixed effect, while the third model further includes logarithmic age as an additional random slope (see Table 2 for details).

To identify the best-fitting model for the present data, the analyses of the following parameters were undertaken: likelihood ratio test (higher is the value, the better the model), Akaike Information Criterion (AIC), and Bayesian Information Criterion (BIC) (with lower values indicating a better fit), assessed through the ANOVA function (24). After the identification step, model quality was further checked by controlling the linearity and normality assumptions. Linearity was assessed using a Tukey-Anscombe plot, while the normal distribution of random effects and residuals was evaluated using Q-Q plots. The Q-Q plot showed that the residuals were normally distributed, as evidenced by the predicted values aligning closely along a diagonal straight line across the standardized residuals (25).

The next step consisted in creating general reference values based on the best-fitted linear mixed model. For easier and practical interpretation of theses references, data were back-transformed to the original scale. Using the model estimation parameters, typical development patterns (mean trajectory, ±1 and ±2 standard deviations from the intercept) were plotted to visually assess the relationship between CA and performance. This relationship was expressed for the mean trajectory using the following equation:

$$sprint\;performance=\;e^{est\;intercept}*CA^{est\;slope}$$

With: est intercept: estimate of the fixed intercept

est slope: estimate of the fixed slope

To complete the reference values, the following typical development patterns *±*1 and ±2 *standard deviations from the intercept* were defined adding ±1 respective ± 2 standard deviations of the random intercept of the grouping factor id to the estimate of the fixed intercept.

### Individual forecasting model

In a subsequent analysis, the individual sprint performance results were plotted against the established reference values to visually depict each athlete's development trajectory. Forecasting models for each athlete were then derived by extracting the individual coefficients (intercept and slope) from the optimal mixed effects model. These coefficients were incorporated into the predictive equation, generating a tailored model that includes both fixed and random effects, thereby enabling the prediction of the athlete's future performance trajectory.

To assess the athlete's performance development relative to the overall group, percentile ranks were determined. Initially, the percentile rank of the individual intercept, which indicates the athlete's initial performance level, was calculated using the LMM. The same approach was applied to calculate the percentile rank for the individual slope, representing the athlete's performance progression over time. This method, inherent to the LMM, allows for the assessment of performance development across time rather than a singular performance point, as is typical with the LMS method.

### Bootstrapping for prediction uncertainty

The accuracy of the predicting performance development is highly dependent on both the complexity of the model and the number of data points available for each athlete. To provide a robust assessment of this prediction, we used a bootstrapping approach to quantify the uncertainty surrounding individual performance predictions (26). Specifically, 1,000 bootstrap samples were drawn from the original data set. For each bootstrap sample, the mixed-effects model was re-estimated, allowing for the recalibration of individual-specific intercepts and slopes. These recalibrated estimates were then used to generate predicted performance scores across the CA range. The resulting distribution of predictions at each CA allows to calculate 95% confidence intervals, providing a detailed measure of uncertainty. These confidence intervals were then overlaid on the individual prediction curves, providing a clear visualization of the potential variability in future performance predictions.

## Results

### Dataset overview

This study analyzed 41'123 race results from 8,732 female 60 m sprint runners aged between 6 and 15 years in Switzerland, covering competitions held between 2006 and 2021.

### Comparison: empirical percentile curves vs. mixed model approach

#### Empirical percentile curves

**Figure 1:**
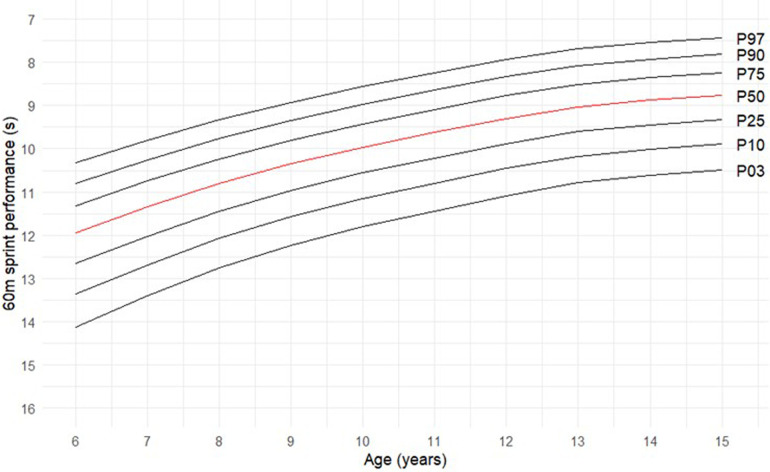
Empirical percentile curves elaborated with the LMS method for the 60 m sprint.

Using the LMS method with the GAMLSS function in R Studio, empirical percentile curves were generated for the dataset. These curves represent the 3th, 10th, 25th, 50th, 75th, 90th and 97th percentiles of performance over 60 m sprint across the different age groups (see Figure 1).

The analysis showed a clear trend of improving performance (faster times) with age for all the percentiles. For example, the median performance time (50th percentile—P50) decreased from 11.35 s at age 7 to 8.75 s at age 15. The percentage improvement is greater in the younger age categories [from 6 years to 7 years −0.60 s (−5.0%)] than in the older ones [from 14 years to 15 years −0.12 s (– 1.3%)]. The percentile curves provided reference values for evaluating individual athlete performance. Athletes performing at predefined percentiles (e.g., the 97th percentile) can be identified as exceptional performers compared to their peers.

#### Mixed model approach

##### Model comparison

According to the linear mixed effect model comparison analysis, the results indicated that Model 3 (CA_SLOPE) (Table 1) provided the best fit for the data [lowest AIC value (−152,493), *χ*^2^ = 3,357,4, *p* < 0.01]. Assumptions for model quality (linearity and normal distribution) were met, supporting the validity of our linear mixed model (For details of the model comparison analysis, see appendix). The development of the sprint performance over 60 m is best explained by the chronological age, including a subject-specific deviation from the overall relationship allowing the slope to vary by subject (Table 2). The addition of log(CAdiff) as random slope induces a supplementary significant and negative effect (beta = −0.18, 95% CI [−0.18, −0.18], t(41,117) = −255.44, *p* < .001; Std. beta = −0.63, 95% CI [−0.64, −0.63]) compared with the two other models. By specifying a random intercept for each individual, the fact that everyone's results may be correlated (for example, a fast individual today is probably fast tomorrow) is considered, which is highly probable in this context.

**Table 1:** Description of the mixed models.

Number	Name	Equation
Model 1	SPRINT_0	LMER (log(performance_sek)∼1 + (1 | id)
Model 2	CA	LMER (log(performance_sek)∼log(CAdiff) + (1 | id)
Model 3	CA_SLOPE	LMER (log(performance_sek)∼log(CAdiff) + [log(CAdiff) | id]
Model 1	SPRINT_0	log(performance_sek_ij_) = *β*0 + b0_j_ + ɛ_ij_
Model 2	CA	log(performance_ sek_ij_) = β0 + β1 log(CAdiff_ij_) + b0j + ɛ_ij_
Model 3	CA_SLOPE	log(performance_ sek_ij_) = (β0 + b0_j_) + (β1 + b1_j_) log(CAdiff) + ɛ_ij_

**Table 2:** Details of the best fitting model.

Predictors	log(performance sek)		
	Estimates	CI	*p*
(Intercept)	2.5899	2.5873–2.5926	<0.001
CAdiff [log]	−0.1775	−0.1789 – −0.1761	<0.001
Random effects			
*σ* ^2^	0.0007		
*τ*_00__id_	0.0090		
τ_11__id.log(CAdiff)_	0.0019		
*ρ*_01__id_	−0.84		
ICC	0.8204		
N _id_	8,732		
Observations	41,123		
Marginal R^2^/conditional R^2^	0.5902/0.9264		

*σ*^2^, Residual variance; *τ*_00__id_, variance of the random intercepts across individuals; *τ*_11__id.log(CAdiff)_, variance of the random slopes for log(CAdiff) across individuals (id); *ρ*_01__id_, correlation between the random intercepts and random slopes; ICC, Intraclass Correlation Coefficient; N _id_, Number of id.

##### Benchmarks

**Figure 2:**
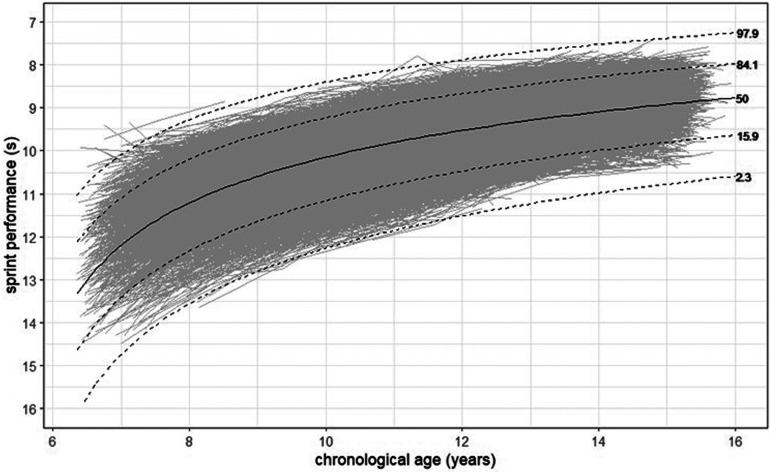
Back transformed data to normal scale with individual models.

In Figure 2, the plot shows typical development patterns for 60 m sprint performance from the best fitted LMM. The black solid line symbolizes the mean performance development (50th percentile). The dashed lines represent the deviations (± 1 SD and ±2 SD) to the mean performance development (i.e., the 2.3th, 15.9th, 84,1th, 97,9th percentiles). The gray lines represent each individual model. For a more practical interpretation, the plot is represented back-transformed to the normal scale.

### Performance prediction

**Figure 3:**
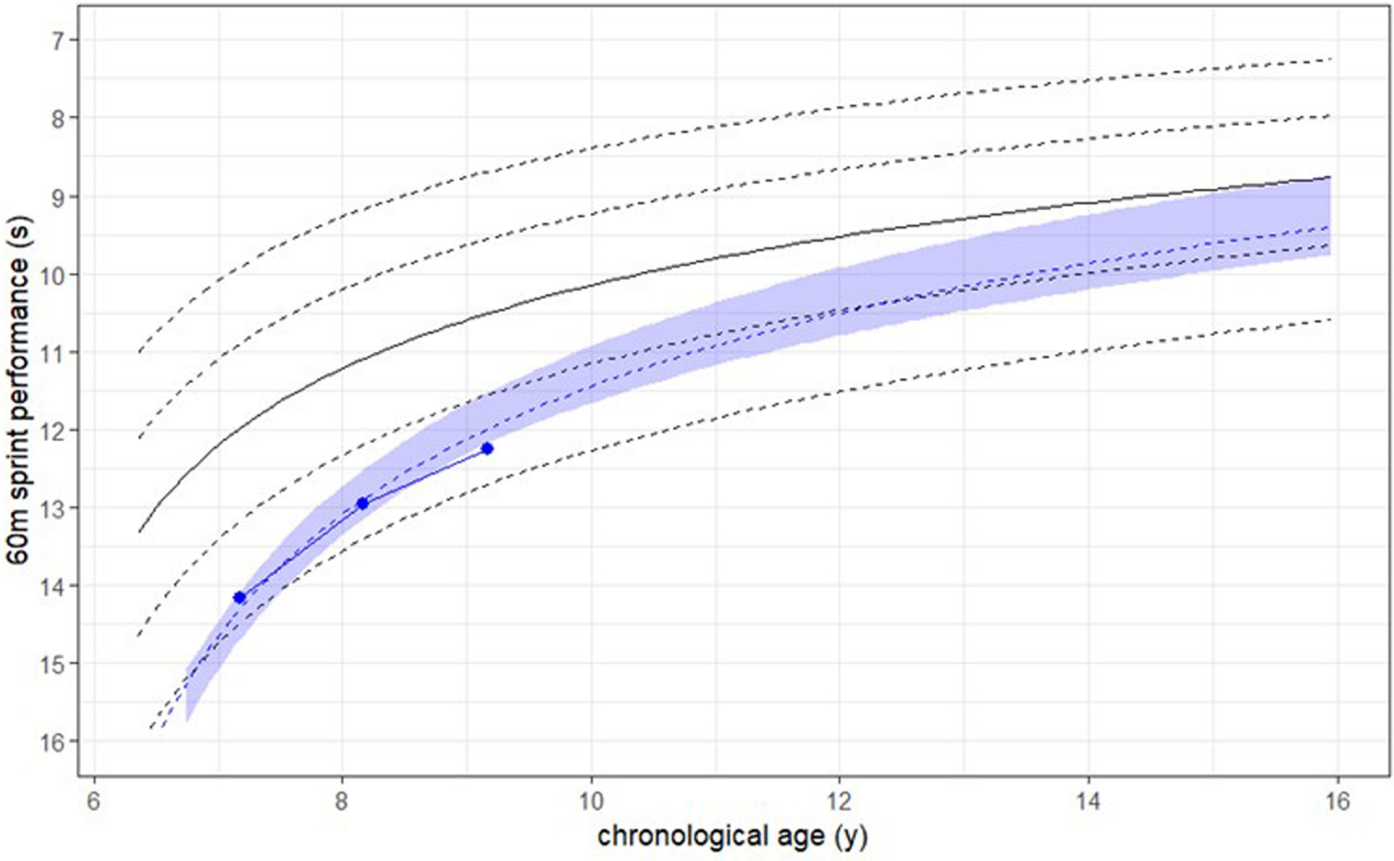
Representation of competitions’ performances of a case athlete example (blue dots) with her performance prediction (dashed blue line) and the respective prediction's accuracy (light blue). Black lines represent the global performance development from the overall population.

The mixed model's ability to predict future performance was evaluated using the longitudinal data. Individual performance trajectories were plotted, and future performance was forecasted based on the model. As the prediction is dependent on model's complexity and the number of existing datapoints for the considered athlete, an accuracy of the prediction was calculated with a bootstrapping approach. The accuracy is shown in Figure 3 for an example case of an athlete, with the light blue area.

### Interpretation of individual trajectories

Individual performance trajectories revealed that athletes with higher initial performance levels showed less improvement over time compared to those with lower initial performance levels. This was evidenced by a negative correlation between random intercepts and slopes (r = −0.84).

In Figure 3, the individual model of a case athlete example (dashed blue line) and her actual performance results (solid blue line) are depicted. By considering the individual athlete's data, both the intercept, which describes the initial performance level, and the slope, which represents the development trajectory, were extracted and percentile ranks were calculated. This approach allows the athlete to be positioned relative to the overall group and is of great interest for long term athlete's development. For example, the analysis indicated that the athlete example depicted in blue had an initial performance level situated at the 14,6th percentile. In comparison to the overall group, this athlete exhibited a very good progression in performance improvement, as reflected by a result at the 97,9th percentile. This comparison highlights the variability in both starting performance levels and developmental trajectories among athletes.

### Practical tool

**Figure 4:**
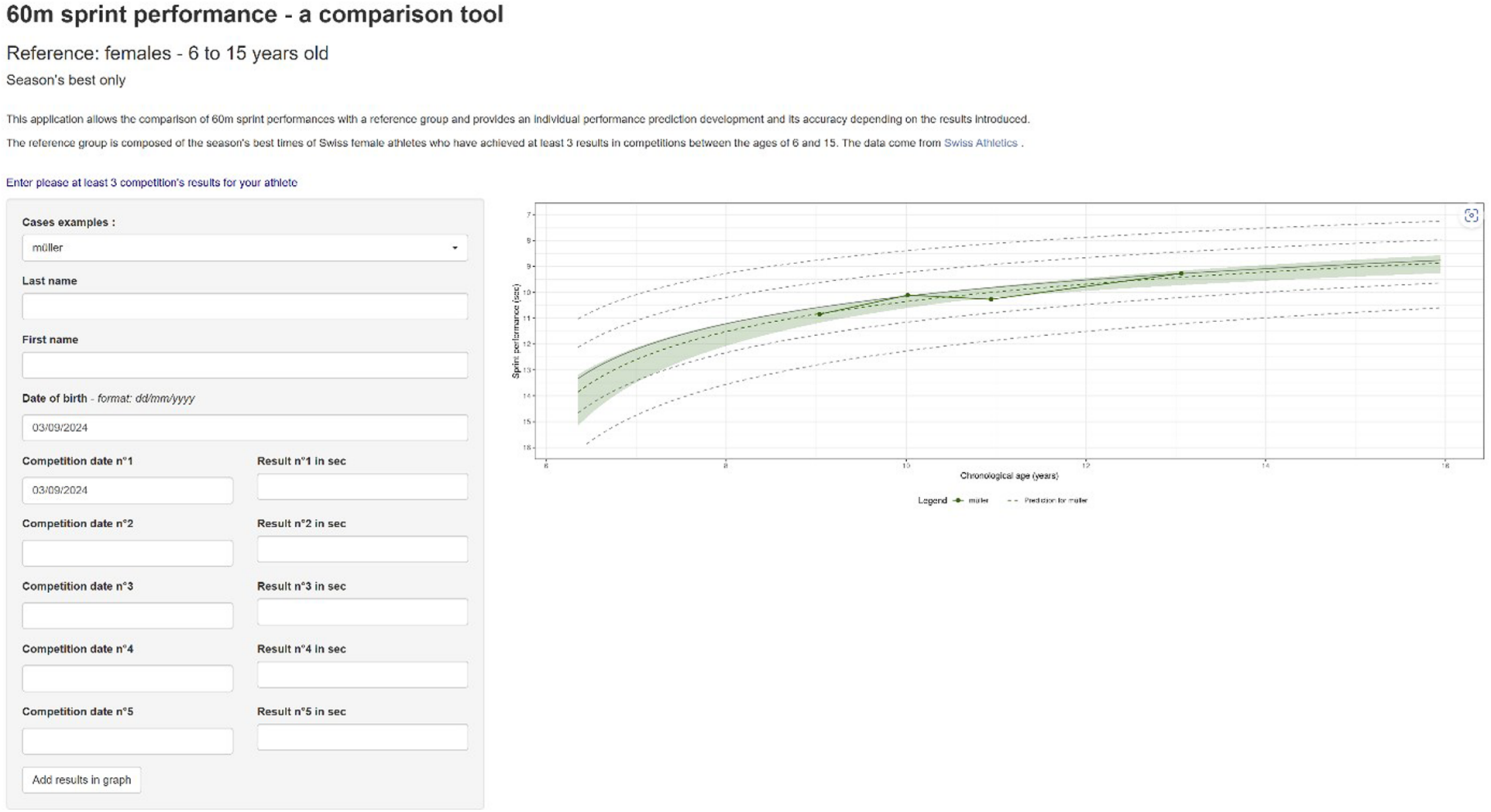
Practical tool for performance prediction.

A shiny app (https://baspo-ehsm-tw.shinyapps.io/sprint_60m_w/) was developed to assist coaches and athletes in predicting future performance (Figure 4). Based on the least three season's best times, the tool predicts performances for the next seasons. As reference values, typical development patterns of the population obtained from the LMM are represented in the background.

## Discussion

The primary objective of this study was to provide age-specific reference values using percentile curves for 60 m sprint performance and to develop a prediction model and a practical software tool for predicting future performance development. Mixed models were able to deal with the complexities of such longitudinal sports data, such as multiple dependent observations and unbalanced data sets. Compared to traditional ANOVA, this approach can provide comprehensive benchmarks and predictive models for performance development.

The results indicated that the best-fitting model included log-transformed chronological age as a fixed effect and both a random intercept and slope for each athlete, demonstrating that these variables significantly impact performance development. This finding is supported by existing literature, which suggests that individual growth patterns and the ability to handle multiple performance observations are crucial for accurate performance prediction (27, 28). This aligns with the findings by Newans et al. (16), who highlighted that traditional repeated measures ANOVA would exclude a significant portion of data, thus limiting the analysis. In this study, mixed models allowed us to include all available data points, regardless of missing data, which is a common occurrence in sports due to factors such as injuries and team selection. The mixed model analysis provided detailed individual performance trajectories, allowing for precise benchmarking and a realistic performance prediction. Athletes with higher initial performance levels (higher positive intercept) exhibited lower positive slopes, indicating less room for improvement. This inverse relationship between initial performance and improvement potential aligns with previous studies in sports science (17). The ability to predict future performance based on individual trajectories is a significant advancement, offering practical tools for coaches and sports scientists to set realistic training goals and can help to identify promising young athletes.

International comparison: article de Tonnessen et al. 2015: only the 100 best athletes from 11 to 18 years old over 60 m (similar progression in performance).

### Methodological considerations

Mixed models offer several advantages over traditional ANOVA, particularly in the context of longitudinal data analysis. They provide greater flexibility in handling missing data, incorporate both fixed and random effects, and allow for the inclusion of multiple predictors. This makes them particularly suited for sports science research, where data heterogeneity and non-standardized measurement intervals are common challenges (29). Furthermore, in our case, the linear relationship between the dependent variable and the explanatory variable obtained through logarithmic transformation makes data analysis more accessible. It may be that, by analyzing other relationships and/or integrating more parameters into the model, the relationship is no longer linear, necessitating the application of more complex mixed models. Making practical application less affordable.

The application of mixed models in our study allowed for a more nuanced understanding of performance development. By explicitly modeling time and accommodating individual variability, mixed models provided insights that would not be possible with traditional methods. For instance, the ability to model individual trajectories and predict future performance offers a significant practical advantage for talent identification and development programs.

### Practical implications

The model provides a robust framework for benchmarking athletes’ performance development relative to their peers, enabling coaches to identify talent and athletes with exceptional potential. Furthermore, the predictive tool facilitates the assessment of individual performance trajectories and the establishment of realistic, data-driven performance goals. By leveraging this tool, coaches can evaluate training effectiveness and detect critical periods that require attention, such as declines in performance, stagnation, or significant improvements. Identifying these patterns creates opportunities for meaningful dialogue with athletes to uncover and address the underlying causes of deviations from expected trajectories. By incorporating a limited yet impactful set of explanatory variables, the current model offers a streamlined and practical approach to understanding factors influencing performance development. For example, a declining trajectory might signal the need for modifications to training loads or recovery practices, while surpassing age-specific benchmarks could indicate readiness for more advanced challenges. These practical applications highlight the value of integrating advanced statistical models and longitudinal data into athlete development, bridging the gap between research insights and actionable coaching practices. Our findings emphasize the importance of continuous monitoring and assessment of athletes’ performance over time. By utilizing longitudinal data and advanced statistical methodologies, coaches and sports scientists can make evidence-based decisions that enhance athlete development, improve training outcomes, and optimize resource allocation.

### Limitations and future directions

Despite the strengths of our study, some limitations warrant consideration. The reliance on existing competition performances introduces potential biases related to competition level, age groups, and specific timeframes. Additionally, the retrospective nature of the analysis and the treatment of outliers could influence the observed performance trajectories. Future research should aim to combine retrospective and prospective data collection with standardized protocols and databases to provide a more comprehensive understanding of athlete development. While the principles of longitudinal performance tracking are broadly applicable and unspecific to sex, physiological and developmental differences between females and males influence performance trajectories. Future studies should therefore establish percentiles and provide the predictive tool for young male athletes. Additionally, sex comparisons may reveal interesting insights into performance development, hence may identify possible specific or generalizable trends. As shown in this study the LMM provides improved prediction of athletes’ performance. Future research should expand this approach to include additional key performance parameters and biological age, which were not considered in the current study. In addition, mixed models should be applied in different sports, age groups, and both genders to validate and extend the results of this research.

## Conclusion

Our study provides performance trajectories and benchmarks using classic LMS percentiles and mixed models, which help to overcome multiple dependent observations and unbalanced datasets in longitudinal performance data and provide a predictive model for future performance. The results provide valuable insights into the complexities of talent development and highlight the importance of using appropriate statistical methods for continuous assessment and benchmarking. The benchmarks and predictive models generated by this research provide practical tools for sports practitioners. The tool can help predict future performance based on individual trajectories, which is a significant advance for coaches and sports scientists. Further research building on this foundation can enhance our understanding of athlete development across different sports and will improve evidence-based practice in sport.

## Data Availability

The original contributions presented in the study are included in the article/Supplementary Material, further inquiries can be directed to the corresponding author.
