# Supplementary material for: Longitudinal performance trajectories of young female sprint runners: a new tool to predict performance progression
Source: Front Sports Act Living. 2024 Dec 23;6:1491064. doi: 10.3389/fspor.2024.1491064 (PMC11700731; doi:10.3389/fspor.2024.1491064)

## Supplementary material

Model description

|  |  |  |  | Fixed effect | Random effects | | |
| --- | --- | --- | --- | --- | --- | --- | --- |
| Model Number | Model specification | Model Name | Dependent variable | Fixed effect | Intercept | Slope |  |
| 1 | random intercept model | SPRINT_0 | log(result_sek) | constant | Individual of each id | - |  |
| 2 | Random intercept and fixed effect model | CA | log(result_sek) | Log(CAdiff) | Individual of each id |  |  |
| 3 | Random and fixed effect | CA_slope | log(result_sek) | Log(CAdiff) | Individual of each id | Log(CAdiff) |  |

1. **Detailed Linear mixed model comparison results**

1.1 model description

Model 1 (SPRINT_0):

| Effect-type | Variable | *b* | *SE b* | 95 % CI | p |
| --- | --- | --- | --- | --- | --- |
| Fixed Effect | Intercept | 2.282 | 0.001 | 2.280-2.283 | < 0.001 |
| Random Effects | Intercept | 0.064 |  | 0.063-0.066 |  |
|  | Residual | 0.069 |  | 0.068-0.070 |  |

*B: beta, estimates; SE standard error; 95% CI: 95% confidence intervals; df: degree of freedom; t: test statistic; p: p values for the coefficients*

Model 2 (CA):

| Effect-type | Variable | *b* | *SE b* | 95 % CI | *t* | p |
| --- | --- | --- | --- | --- | --- | --- |
| Fixed Effects | Intercept | 2.577 | 0.001 | 2.575 – 2.579 | 2701.0 | <0.001 |
|  | Log(CA_mindiff) | -0.1714 | 0.0004 | -0.1722 - -0.1705 | -386.4 | <0.001 |
| Random Effects | Intercept | 0.052 |  | 0.050 – 0.052 |  |  |
|  | Residual | 0.0297 |  | 0.0294 – 0.0299 |  |  |

Model 3 (CA_slope):

| Effect-type | Variable | *b* | *SE b* | 95 % CI | *t* | p |
| --- | --- | --- | --- | --- | --- | --- |
| Fixed Effects | Intercept | 2.589 | 0.001 | 2.587 – 2.593 | 1885.7 | <0.001 |
|  | Log(CA_mindiff) | -0.17775 | 0.0001 | -0.1789 - -0.1761 | -255.4 | <0.001 |
| Random Effects | Intercept | 0.095 |  | 0.092 – 0.097 |  |  |
|  | Log(CA_mindiff) | 0.044 |  | 0.043 – 0.046 |  |  |
|  | Residual | 0.02566 |  | 0.0253 – 0.0258 |  |  |

1.2 model comparison

| Model number | Model | comparison | *df* | AIC | BIC | -logLik | Χ^2^ | p |
| --- | --- | --- | --- | --- | --- | --- | --- | --- |
| 1 | SPRINT_0 |  |  | -89254 | -89229 | 44630 |  |  |
| 2 | CA | 1 vs 2 | 1 | -149139 | -149105 | 74574 | 59887 | < 0.001 |
| 3 | CA_slope | 1 vs 3 | 3 | -152493 | -152441 | 76252 | 63245 | < 0.001 |
|  |  | 2 vs 3 | 2 |  |  |  | 3357.4 | < 0.001 |

***df****:degrees of freedom;* ***AIC****: Aikake Information Criterion;* ***BIC****: Bayesian Information Criterion; Χ^2^: Chi-square*

**Quality of the best fitted model:**

**Linearity of residuals**

The Tukey-Anscombe plot (Figure xx) was used to assess the linearity assumption. The residuals were evenly spread around zero across all fitted values. This suggests that the linearity assumption was adequately met.


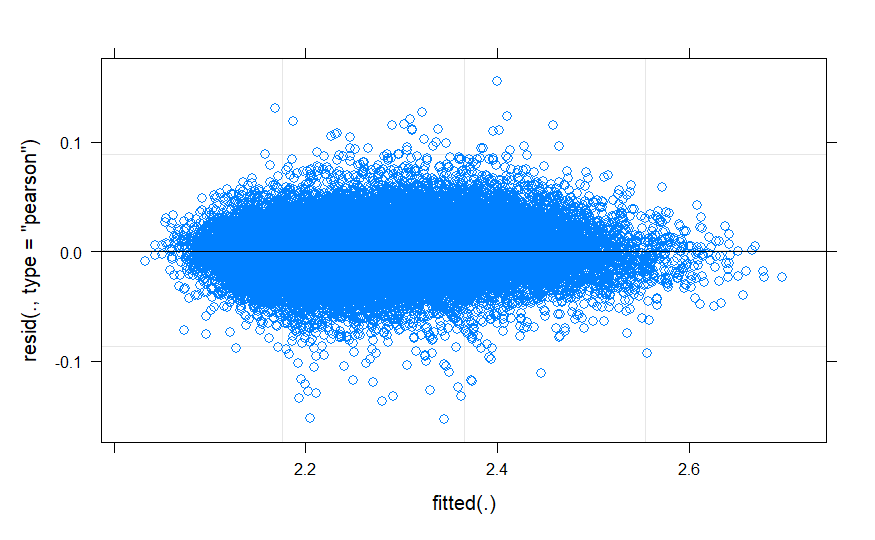


**Normal Distribution of Random Effects and residuals**

Normality of the random effects was examined by constructing Q-Q plots for the intercept variable (id) and the slope variable (CA_mindiff). The same plot was done for the residuals (Figure XX). The Q-Q plot for random intercept and slope showed that the random effects were approximately normally distributed, as the points closely followed the theoretical normal distribution line. The residuals closely followed the theoretical normal distribution line, with slight deviations at the extremes. This indicates that the residuals were approximately normally distributed.


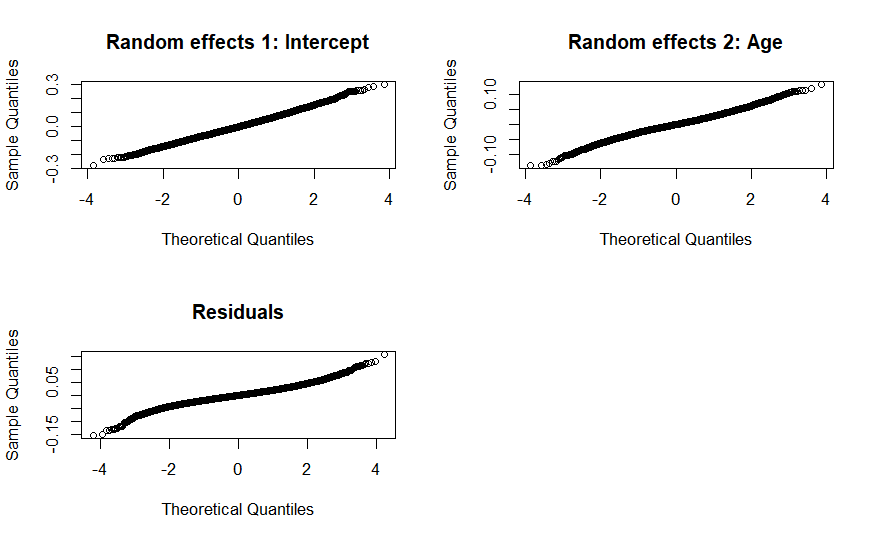

Supplement: Supplementary file 1 [file Table1.docx]
